# Real-time observation of polymerase-promoter contact remodeling during transcription initiation

**DOI:** 10.1038/s41467-017-01041-1

**Published:** 2017-10-27

**Authors:** Cong A. Meng, Furqan M. Fazal, Steven M. Block

**Affiliations:** 1 Department of Chemistry, Stanford University, Stanford, CA 94305 USA; 2 Department of Applied Physics, Stanford University, Stanford, CA 94305 USA; 3 Department of Biology, Stanford University, Stanford, CA 94305 USA; 4 Present Address: Stanford University School of Medicine, Stanford, CA 94305 USA

## Abstract

Critical contacts made between the RNA polymerase (RNAP) holoenzyme and promoter DNA modulate not only the strength of promoter binding, but also the frequency and timing of promoter escape during transcription. Here, we describe a single-molecule optical-trapping assay to study transcription initiation in real time, and use it to map contacts formed between σ^70^ RNAP holoenzyme from *E. coli* and the T7A1 promoter, as well as to observe the remodeling of those contacts during the transition to the elongation phase. The strong binding contacts identified in certain well-known promoter regions, such as the −35 and −10 elements, do not necessarily coincide with the most highly conserved portions of these sequences. Strong contacts formed within the spacer region (−10 to −35) and with the −10 element are essential for initiation and promoter escape, respectively, and the holoenzyme releases contacts with promoter elements in a non-sequential fashion during escape.

## Abstract (web summary)

Contacts between RNA polymerase and promoter DNA modulate the strength of binding and the frequency of promoter escape during transcription. Here, the authors describe a single molecule optical-trapping assay to study transcription initiation and observe the dynamic remodeling of enzyme contacts in real time.

## Introduction

The initiation of transcription is one of the most extensively regulated steps in gene expression^1, 2^. In bacteria, the complex responsible for this critical step is the RNA polymerase holoenzyme, comprised of the RNA polymerase (RNAP) core enzyme in combination with a single copy of a specificity factor, sigma (*σ*). The RNAP holoenzyme is able to search for, and bind, promoter DNA, thereafter forming an RNAP-promoter closed complex (RP_c_). The bound holoenzyme then unwinds ~ 12–14 base pairs (bp) of double-stranded DNA (dsDNA)^1^ to form the RNAP-promoter open complex (RP_o_). The open complex undergoes a process of abortive initiation, involving repeated episodes of DNA “scrunching,” during which the RNAP remains more-or-less stationary on the promoter, as it repeatedly unwinds and pulls in a segment of downstream DNA, while synthesizing a series of short RNA oligomers, 3–11 nt in length^3–5^. The RNAP enzyme eventually escapes the promoter region, transitioning to its elongation phase, which is characterized by the formation of an elongation complex (EC) and the processive production of a longer, nascent RNA.

The promoter region contains a number of consensus sequence elements specifically recognized by RNAP. Two well-studied hexameric sequences, the −10 and −35 elements^6^, as well as a third consensus sequence, called the extended −10 element^7^, are known to make direct contacts with regions 2, 4, and 3 of *σ* factor, respectively^8^. The UP element, a sequence located upstream of the −35 element and rich in A/T, is known to stimulate transcription by binding the C-terminal domain of the α subunit of RNAP (the α-CTD)^9–11^. Contacts mediated between promoter elements and the RNAP holoenzyme modulate the frequency of transcription initiation, and thereby regulate gene expression^12^.

Previous structural, biochemical, and biophysical studies^13–17^ have provided snapshots of holoenzyme-promoter contacts, and a variety of single-molecule approaches have proved useful in dissecting additional mechanistic and kinetic details of initiation in prokaryotes^3, 4, 18–20^ and eukaryotes^21^, but key questions remain. In particular, how does RNAP remodel its contacts with the promoter DNA during the initiation phase, ultimately leading to the formation of the EC?

Here, we describe a single-molecule optical-trapping assay^22^ that can probe the double-stranded DNA (dsDNA)-stabilizing contacts formed by the initiation complex, as well monitor the progress of transcription initiation in real time. Using the assay, we identified strong binding contacts between the *E. coli σ* ^70^ RNAP holoenzyme and promoter DNA sequences in both the closed (RP_c_) and open (RP_o_) complex states. We find that a strong contact within the so-called “spacer region” of the promoter, situated between the well characterized –10 and –35 elements, is essential to the initiation process, and that the RNAP holoenzyme releases its contacts with various promoter elements in a non-sequential order during promoter escape.

## Results

### Structural determinants of the initiation process

**Fig. 1:**
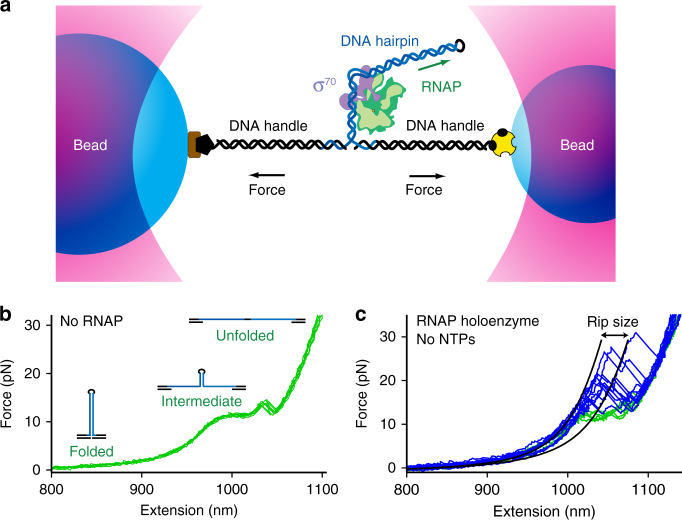
The optical-trapping assay. **a** Cartoon illustrating the “dumbbell” assay for transcription initiation (not to scale). Two polystyrene beads are held in individual optical traps. DNA handles are attached to the two beads via biotin-avidin (yellow) and digoxiginin-antidigoxiginin (brown) linkages. A DNA hairpin (blue) carrying a promoter region in its stem is annealed to the handles (black) on both ends. RNAP holoenzyme binds the promoter and transcribes towards the hairpin loop (black). **b** Representative FECs of the hairpin bearing a T7A1 promoter, extending from −56 to +20, in the absence RNAP. **c** FECs collected with holoenzyme present, with either RNAP bound (blue) or not bound (green). All data were obtained using the same promoter construct. WLC fits to the data before and after a rip in a representative record (black lines)

To study initiation, we developed a hairpin unzipping assay that is conceptually similar to assays previously used to interrogate protein-nucleic-acid contacts by single-molecule force spectroscopy^23–25^. The assay consists of two polystyrene beads, each held in a separate optical trap^26^, and attached to dsDNA handles flanking a single DNA hairpin that carries a promoter with a transcription initiation site (Fig. 1a). This site consists of a promoter sequence extending from positions −56 to +20 (relative to the transcription start site, TSS, defined as position +1), derived from the wild-type T7A1 promoter. In our first experiments, the promoter was orientated such that the direction of transcriptional motion was towards the hairpin loop, referred to as “co-directional” pulling (Fig. 1a, green arrow). The optical-trapping apparatus allowed us to apply controlled loads to the base of the hairpin, via the handles, which could be used to unzip it mechanically. All experiments were carried out at 26 ± 1 °C.

When loads were applied to the promoter hairpin in the absence of the RNAP holoenzyme, two “rips”—that is, abrupt increases in the tether extension—were observed in the resulting force-extension curves (FECs), each corresponding to a partial unzipping of the duplex stem structure (Fig. 1b). The distinct rips indicate the existence of three states (folded, intermediate, and unfolded)^27, 28^ during unzipping of this long (76 bp) hairpin.

When RNAP holoenzyme was introduced into the assay, the FECs instead displayed multiple rips at high loads, in excess of 15 pN (Fig. 1c, blue). These events correspond to the release of contacts associated with the holoenzyme binding to specific hairpin sequences. During the elapsed interval between successive pulls in these experiments (~ 30 s), the holoenzyme has sufficient time to bind the T7A1 promoter in its closed state, RP_c_, and transition to the open complex, RP_o_, based on the lifetime measured for RP_c_, which is under 20 s^17, 29, 30^. The formation of the open complex under our experimental conditions was confirmed in a separate experiment using digestion by potassium permanganate^31^ (Supplementary Fig. 1). The observed rips therefore occur at positions where contacts are broken from the RP_o_ and RP_c_ states during hairpin unzipping. By fitting the FECs with a double worm-like-chain (WLC) model (Fig. 1c, black curves)^27^, we determined the opening distances associated with individual rips, which were subsequently mapped to specific nucleotide positions within the promoter sequence, relative to the TSS (+1), thereby generating a high-resolution DNA contact map from the trailing edge to the leading edge of the RNAP holoenzyme.

**Fig. 2:**
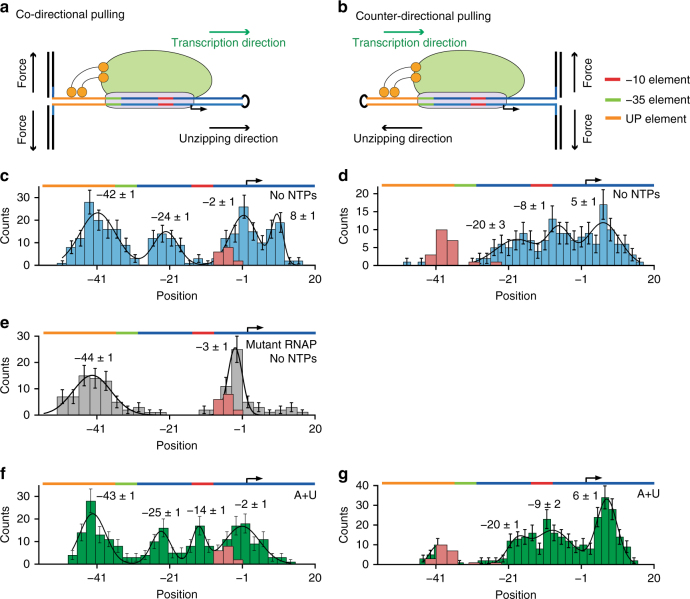
Mapping RNAP holoenzyme-promoter contacts. **a**, **b** Cartoon illustrating the assay geometry. A hairpin stem bearing the T7A1 promoter, extending from positions −56 to +20, was used for both co-directional and counter-directional studies, with a tetraloop (black) at one end and dsDNA handles attached to other. RNAP (green ellipse) with σ^70^ (purple) recognizes the promoter, including the −10 (red), −35 (green), and UP (orange) elements, and initiates transcription from the TSS (right-angle arrow). Optical forces can be applied to break RNAP-promoter contacts in either transcriptional direction. **c**, **d**, **f**, **g** Mapped distributions of the “rip” positions for wild-type RP_o_, with (green) or without (blue) ATP + UTP present. Hairpin intermediate states are also shown (pink). The promoter axis (colored) is shown on top for reference. **e** Mapped distributions of rips with a strand-opening-deficient mutant RNAP (no NTPs)

To investigate further the holoenzyme-promoter contacts, we created a second hairpin construct, based on the identical stem sequence, but with the orientation of the promoter reversed, to create a “counter-directional” pulling geometry. With this construct, the contacts are released in the reverse order, from the leading edge towards the trailing edge of RNAP. Both the co-directional and counter-directional assays are illustrated in Fig. 2a, b, and the positions of the associated rips are compared in Fig. 2c–g. The positions of rips obtained in the absence of holoenzyme are included for reference (Fig. 2c–g, pink).

With holoenzyme present, but in the absence of added nucleoside triphosphates (NTPs), the co-directional pulling assays revealed contacts located at positions −42 ± 1, −24 ± 1, −2 ± 1, and +8 ± 1 (mean ± S.E.M.; *N* = 284 rips from 18 molecules; Fig. 2c). Counter-directional pulling assays performed under otherwise identical conditions identified the contacts observed previously (at −20 ± 3, +5 ± 1), plus an additional contact at −8 ± 1 (mean ± S.E.M., *N* = 162 rips from 15 molecules; Fig. 2d). Similar co-directional pulling experiments were carried out to explore contacts formed by RP_c_, by using a strand-opening-deficient mutant RNAP holoenzyme that is unable to form a transcription bubble, and thus gets trapped in the RP_c_ state^30^. Unzipping results for RP_c_ showed contacts near positions −42 (−44 ± 1) and −2 (−3 ± 1) (*N* = 123 rips from 13 molecules; Fig. 2e), but wild-type contacts at −24 to −20, and +5 to +8 were absent (Fig. 2c). Taken all together, the experimental results indicate that stabilizing contacts in the closed form are located near positions −42 and −2, and upon transition to RP_o_, additional contacts are formed in −24 to −20 and +5 to +8.

The contact near the trailing edge of RNAP, at position −42, suggests a tight interaction between RNAP and the UP element in both RP_o_ and RP_c_ (Fig. 2c, e). Following up on this possibility, we did not observe binding of the holoenzyme to a shorter, 61-bp hairpin construct that excluded 15 bp (−56 to −42) from the upstream promoter sequence, which forms a part of the UP element. Deleting this portion of the UP element abolished binding, consistent with previous studies which reported that this sequence greatly stimulates transcription^9, 11^. Similarly, the strong interactions formed at the RNAP leading edge (+5 to +8) in RP_o_ lend support to a previous suggestion that these contacts allow for the proper closure of the RNAP clamp, in order to position the enzyme for subsequent catalysis^32^. Moreover, our data are consistent with a previous study showing that the RNAP clamp closes upon the transition from RP_c_ to RP_o_^19^, resulting in tighter binding to DNA elements downstream of the TSS in RP_o_. Similarly, the contact we observed near position −2 is consistent with crystallographic evidence showing that the RNAP holoenzyme-promoter makes contacts from positions −4 to −2, which constitute the core-recognition element (CRE)^16^. The contacts from −12 to +2 are also supported by previous permanganate-footprinting experiments of RNAP on T7A1^29^.

In contrast to the three regions described above, which have previously been implicated using either biochemical or structural approaches, the contacts we detected from −24 to −20 have not been well studied, nor has any associated consensus sequence been identified. This promoter sequence covers the spacer region between the −10 and −35 elements. Yuzenkova et al.^33^ proposed that this spacer region forms sequence-specific contacts with the RNAP β′ subunit, and hypothesized the existence of a novel class of promoters that may rely upon this interaction. A recent study^34^ performing cross-linking experiments found some evidence for contacts between the β′ subunit and the −21 and −20 positions on the template strand in RP_o_, as did biochemical experiments carried out on the T7A1 promoter (−23 to −21 protected)^17^.

To confirm the existence of contacts in this region, we explored contacts formed in the presence of the first two initiating nucleotides (ATP, UTP), which are thought to stabilize the open complex. In both pulling geometries (*N* = 236 rips from 14 molecules; Fig. 2f; *N* = 297 rips from 13 molecules; Fig. 2g) we observed contacts at the same locations, within error, as those previously determined for RP_o_ in the absence of nucleotides, but also observed a stabilizing contact located at position −14 ± 1 (Fig. 2f). Determination of the force required to break the first contact during unzipping (Supplementary Fig. 2a–c) revealed that a higher force was necessary to break promoter contacts in the presence of ATP and UTP (>25 pN) in the counter-directional assay (Supplementary Fig. 2b, c). Our results therefore confirm that the first two initiating nucleotides serve to stabilize RP_o_ additionally while preserving previous contacts.

When we examined the forces at which the contacts dissociated, we did not find statistically significant differences in the forces for co-directional pulling experiments across different conditions (Supplementary Fig. 3a). The force required to break the first contact during unzipping was the same, within error, among conditions with ATP plus UTP and with no NTPs (Supplementary Fig. 2a). For counter-directional pulling, we observed only a small increase in the dissociation force when both nucleotides were present, relative to no NTPs (30 ± 2 pN vs. 26 ± 2 pN, respectively; mean ± S.E.M., Supplementary Fig. 3b).

In the presence of all four NTPs, RNAP can escape the promoter and enter the productive elongation phase. When we added NTPs to the co-directional pulling assay (saturating conditions; 1 mM), we no longer observed discontinuous rips in the FECs (Supplementary Fig. 2d). To confirm that the loss of these rips reflected successful transcription initiation, we performed an identical experiment in the presence of rifampicin. Rifampicin inhibits RNAP during the initiation phase after it has incorporated the first 2–3 nt^35^, but it exerts no significant effect on holoenzyme binding. In the presence of rifampicin, we recovered the same rips that had previously been associated with promoter contacts (Supplementary Fig. 2e), confirming the assignment. We interpret the loss of features in the FECs upon the addition of NTPs as resulting from RNAP undergoing successful promoter escape, and thereafter stalling upon reaching the end of the promoter DNA. The polymerase enzyme remains bound to the template strand after unzipping, thereby preventing any subsequent reannealing of the two strands to reform the hairpin, even after the applied force is lowered. This interpretation is supported by a recent study that examined RNAP paused during elongation while bound to dsDNA, which reported that the enzyme remained bound to the template strand even after the separation of the DNA strands by an external force^36^.

### Real-time observation of transcription initiation

**Fig. 3:**
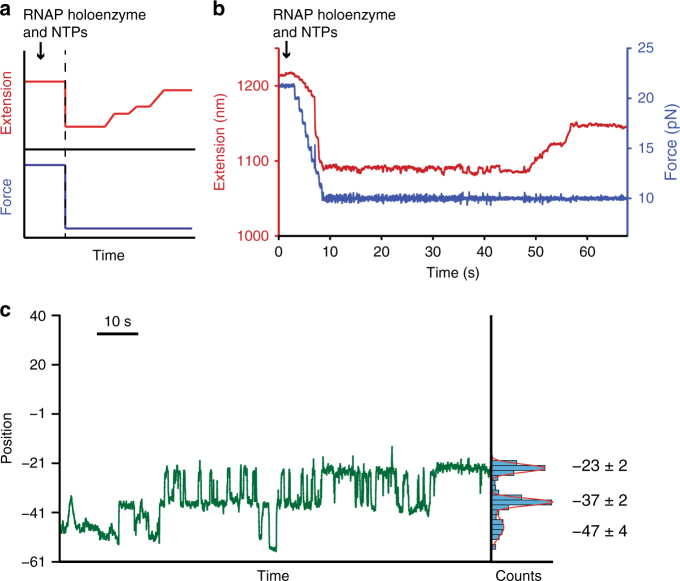
Real-time observations of dsDNA-stabilizing contacts remodeled during transcription initiation. **a** Force protocol for real-time initiation experiments (blue), displayed with a notional data showing RNAP binding and initiation (red). A DNA hairpin is held initially at constant high force, sufficient to keep it unfolded, resulting in a large starting tether extension. RNAP holoenzyme plus NTPs are introduced into the flow chamber (black arrow) while high force is maintained. After the flow stabilizes, the force is lowered below the hairpin opening force (*F*_1/2_, vertical dashed line), and thereafter maintained at ~ 1–2 pN below *F*_1/2_. The hairpin refolds promptly, after which the holoenzyme binds and tether extension increases as RNAP remodels contacts during subsequent transcription. **b** A representative experimental record, showing the external load (blue) and concomitant tether extension (red) for the 40DT template sequence. The initial force, ~ 20 pN, was decreased in a series of steps to ~ 9 pN. After 48 s, transcription led to an increase in tether extension of ~ 50 nm. **c** RNAP holoenzyme position under ~ 11 pN load in the absence of NTPs; the enzyme was introduced shortly after the start of the record. Measured extension (in nm) were converted to nucleotide position relative to the TSS (+1 site) of the promoter

Having used force spectroscopy to identify contacts formed in the binding phase, we next turned our attention to how these contacts get remodeled during the initiation phase. Owing to the dynamic nature of the initiation process, we needed to start data collection before the holoenzyme bound to the promoter, and to monitor transcriptional progress thereafter. The assay we developed is summarized in Fig. 3a, b, with a detailed explanation found in the Methods section. Briefly, a force clamp^26^ was used to maintain constant load on the DNA dumbbell tether throughout data collection. The external force was set to 11–12 pN, a force that is 1–2 pN below the hairpin opening force, permitting a double-stranded promoter region. Subsequent holoenzyme binding and any remodeling of contacts with the promoter DNA lead to unzipping of the dsDNA region, upstream from the RNAP. The conversion from dsDNA to ssDNA leads to nanoscale changes in tether extension, which can be converted directly into position along the hairpin relative to the TSS. We are thereby able to track RNAP binding and subsequent initiation in real time by monitoring the advance of the trailing edge of RNAP along the promoter hairpin.

We first performed real-time studies in the absence of NTPs (Fig. 3c). After RNAP bound, the DNA hairpin was found in one of three partially open states, located at positions −47 ± 3, −37 ± 5, and −22 ± 3 (mean ± S.D., *N* = 5 records). Absent a source of chemical energy (NTPs), the holoenzyme is expected to remain stationary in either a closed or open state. The three states observed therefore result not from the translocation of RNAP, but from the remodeling of contacts following binding. The observed states are consistent, within experimental error, with the contacts at positions −42, and at −24 to −20, that were assigned in our earlier unzipping experiments (Fig. 2). Additionally, the −37 ± 2 state is consistent with RNAP making contact with the −35 promoter element^2, 14^. The fact that contacts upstream of −23 were released reversibly from the open state suggests that these contacts may not be essential for subsequent initiation steps. This notion gains support from a real-time record where an individual holoenzyme lost contacts upstream of the −20 position, yet still underwent promoter escape and a successful transition to the elongation phase when NTPs were subsequently added (Supplementary Fig. 4a). However, we never observed release of the −23 contact prior to NTP addition and subsequent promoter escape, which suggests that this contact, situated within the spacer region, may be required for subsequent initiation events.

**Fig. 4:**
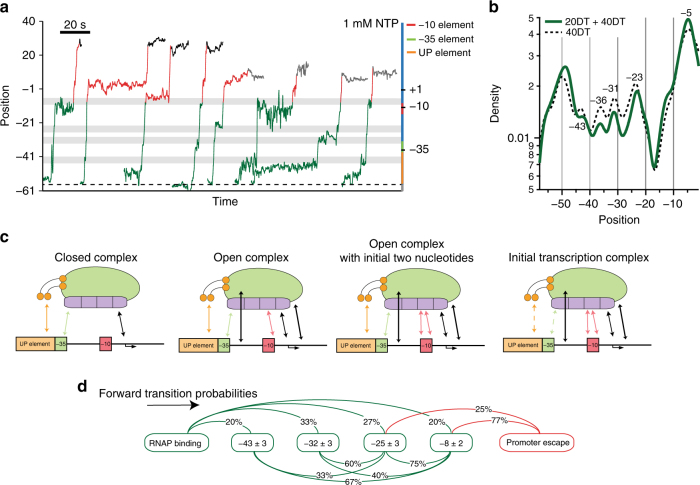
Real-time transcription initiation records. **a** Eight representative records showing RNAP holoenzyme initiation. Left axis: holoenzyme position; measured extensions (in nm) were converted to nucleotide positions relative to the TSS. Right axis: non-template strand promoter sequence, using the color scheme of Fig. 2. The starting base of the hairpin stem is indicated (black dashed line, position −56). The records are color coded with respect to the stages of initiation process: stepwise contact remodeling (green), productive initiation (red), and stalled at the end of the template (40DT template, black; 20DT template, gray). Gray bars indicate the positions of strong contacts. **b** Semi-log plots of the mean density of records obtained in the presence of NTPs on the 40DT template (*N* = 12, black dotted curve), and the 40DT and 20DT templates combined (*N* = 16, green curve). The maxima confirm the assignment of contacts (see text). **c** Summary of the contacts remodeled within the RNAP-promoter complex during transcription initiation. Solid arrows indicate essential contacts; dashed arrows indicate non-essential contacts (arrows are color coded by their respective elements). **d** Transition probabilities in the forward direction (black arrow, computed percentages exclude reversible transitions between states) from RNAP binding to promoter escape (*N* = 44 transitions from 15 molecules, color code same as **a**). The states (rounded squares) were identified based on dwells lasting >1 s, and named according to their positions on the T7A1 promoter

Finally, we performed real-time studies under saturating NTP conditions (1 mM) to examine the initiation process with two different constructs, containing a promoter region followed by template DNA downstream from the TSS, consisting of either 20 bp (construct “20DT”) or 40 bp (construct “40DT”) (Fig. 4a). On these substrates, we expect the RNAP holoenzyme to bind, remodel its dsDNA-stabilizing contacts, and then, following promoter escape, translocate for a distance less than, or equal to, the remaining length of the template (20 or 40 bp). End-to-end distance changes in the assay are therefore generated by two distinct mechanisms: (1) remodeling of RNAP-promoter contacts (green region, Fig. 4a) and (2) translocation of RNAP during transcription (red region, Fig. 4a). In addition to the states that we previously observed in the absence of NTPs (positions −43 ± 3, −32 ± 3, and −25 ± 3; mean ± S.D.; Fig. 4a, gray bars), we observed a state at position −8 ± 2 that was consistent with our previous unzipping data (Fig. 2c–f), indicative of a contact being formed near the −10 element. To confirm these states, we produced probability-density plots of individual records as functions of position (Supplementary Fig. 4b, *N* = 16), and aggregated these to generate a global mean-density plot (Fig. 4b). This plot indicates high probabilities of occupancy at positions that are identical, within error, as those identified above, providing additional confidence in the assignments.

We propose that RNAP escapes the promoter promptly upon the release of contacts near position −8 (Fig. 4a). Consistent with this proposal is the observation that the pause-free velocity of RNAP at the start of elongation is 23 ± 5 nt s^−1^ (mean ± S.D., *N* = 8), comparable to the full, pause-free velocity of ~ 18 nt s^−1^ under similar loads and conditions determined previously^37^. During promoter escape, the motion would therefore consist of contact release, followed by hairpin unzipping to position + 8 ± 4 (mean ± S.D., *N* = 4) on 20DT, or to position + 26 ± 2 (mean ± S.D., *N* = 7) on 40DT, positioning the RNAP trailing edge located at ~ + 8 or ~ + 26, respectively, after stalling at the hairpin loop. The net distance of translocation along the promoter DNA would therefore be 16 ± 5 bp (8 + 8 bp) or 34 ± 3 bp (26 + 8 bp): equal, within experimental error, to the lengths of the downstream DNA sequences (20 and 40 bp, respectively), and consistent with observations. This proposal was further supported by a real-time record where RNAP was first advanced by 29 bp on the 40DT template by supplying three of the four NTPs (Supplementary Fig. 4a; see Methods section). In this case, we observed translocation of 30 ± 3 bp after the release of contacts at position −8. Transcription continued after the addition of all four NTPs, after which RNAP advanced an additional 9 ± 3 bp. Our results across different records, summarized in Fig. 4a–c, highlight the dynamic remodeling of contacts that occurs after RNAP binds its promoter.

## Discussion

In this work, we developed an optical-trapping assay to study RNAP initiation at the single-molecule level. We found that RNAP makes stabilizing contacts with specific elements of the T7A1 promoter, which are subsequently remodeled during the transition to elongation phase. Several of these contacts are well-established and have previously been studied by traditional biochemical or genetic approaches; others have not. The present assay offers a versatile approach that can be straightforwardly adapted to examine contacts with different promoter sequences, as well the effects of different *σ*-factors in modulating those contacts. We anticipate that the assay may be extended to the study of other processive nucleic-acid motors and binding proteins, including those involved in the complex machinery that drives eukaryotic transcription. As a proof of principle, we assembled yeast RNAP II (pol II) in a 32-component, preinitiation complex (PIC) on a hairpin carrying the native promoter, *His4* ^38^, and identified contacts between the PIC and promoter DNA, based upon the series of rips observed at high forces (>25 pN) (Supplementary Fig. 5). The mean force at which the full PIC dissociated (27 ± 1 pN, mean ± S.E.M.) was significantly higher than in the presence of TATA-binding protein alone (17 ± 1 pN).

In the case of the bacterial RNAP holoenzyme-DNA complex, we consistently observed contacts for RP_o_ at positions −42, −24 to −20, −8, −2, and +5 to +8 (Fig. 2) in the absence of NTPs. The majority of RNAP enzymes are likely to be in the open-complex form, having bound DNA and transitioned from RP_c_ to RP_o_ on the timescale of our pulling experiments, consistent with a previous report^17^. Based on the fraction of wild-type RNAP molecules that displayed contacts not otherwise observed in the strand-opening deficient mutant we estimate that at least 46 ± 4% (*N* = 155) were in RP_o_, as a lower bound (Fig. 2c, g).

Of the contacts scored, only those at positions −42 and −2 were observed in RP_c_. The addition of the first two initiating nucleotides (ATP, UTP) induced remodeling changes that generated contacts at −14 (Fig. 2g). Real-time data obtained in the presence of NTPs lent further support for these assignments, while revealing an additional contact in the region of −32 to −35. These assignments are consistent with previous structural studies^14, 16, 34^, hydroxyl-radical footprinting experiments of RP_o_^17, 39, 40^, and a recent study that reported a structure for *Thermus aquaticus* (Taq) RNAP with a full transcription bubble^41^.

The contacts scored in FECs, and the corresponding states observed in real-time records, are summarized in Fig. 4c. The contact at −42 must be essential for initiation, because removing it completely abolished binding in a truncated promoter. The real-time records also reveal a state at −49 ± 2, which was observed both in the absence (Fig. 3b) and presence of NTPs (Fig. 4a, b; Supplementary Fig. 4b). Because we only observed this contact when RNAP was present, we conclude this is the upstream-most contact on our T7A1 promoter template. It may serve to anchor RNAP to the UP element of the promoter via α-CTD elements. This assignment is consistent with the findings of Sclavi et al.^17^, who observed this contact in the absence of NTPs using permanganate-footprinting assays.

Likewise, the contacts within the spacer region of −24 to −20 appear to be indispensable for initiation. The contact at position −8 ± 2 is the final contact upstream of the transcription bubble that gets released upon promoter escape. The average transition time from RNAP binding to promoter escape was 27 ± 15 s (mean ± S.D., *N = *12, 26 ± 1 °C). This duration is consistent with recent biochemical studies that estimated the transition time to be between 12–30 s (the transition from RP_c_ to RP_o_ takes ~ 2–20 s^17^, and that from RP_o_ to promoter escape take <10 s^29^).

Intriguingly, whereas the contacts upstream of position −24, which include the UP and −35 elements, are essential for initial RNAP binding, they do not seem to be required for the subsequent of initiation. In real-time records, RNAP released the contacts from −43 and −32 prior to promoter escape (Fig. 4d). On its face, this observation appears to be inconsistent with one previous single-molecule study that concluded that the trailing edge of the RNAP does not move relative to DNA prior to promoter escape^4^. It seems possible that upstream contacts may be significantly weakened, but perhaps not lost altogether, during early initiation, and therefore continue to anchor the RNAP position weakly.

In real-time records, the states identified correspond to specific sets of contacts made by RNAP and the promoter at the corresponding positions. Taken all together, the records of binding and promoter escape imply a non-sequential ordering of events during the initiation process (Fig. 4a, d). Not every state identified overall was found in each individual record (Fig. 4a, gray bars), and the likely explanation for such “missing” states is that contact remodeling can occur out of order, with downstream contacts occasionally being released prior to upstream ones. Because the real-time assay monitors the position of the trailing edge of RNAP, tether extension changes are scored only when most upstream of a given set of contacts gets released: any prior release of downstream contacts therefore leads to a missing state.

Finally, we observe that the contacts identified here near the canonical promoter regions, such as the −35 and −10 elements, do not perfectly co-locate with the most highly conserved portions of these sequences. An emerging view is that that a fully consensus promoter sequence might, in fact, be undesirable^12^, because an over-abundance of RNAP-promoter contacts would serve to inhibit transcriptional activity by binding too tightly, thereby impeding the transition from RP_o_ to promoter clearance^42, 43^. Presumably, the most efficient promoters evolved to satisfy conflicting constraints, forming sufficiently numerous contacts to ensure proper promoter recognition, but not so many as to inhibit subsequent promoter escape.

## Methods

### Dumbbell preparation

For the single-molecule experiments, we developed a “dumbbell” assay, with each dumbbell comprised of a DNA hairpin carrying the T7A1 promoter connected to two dsDNA handles attached to two polystyrene beads^27, 28, 44^. The base of the hairpin stem ended with an abasic site on each strand (to minimize any possible steric hindrance), and carried a 25-nt, single-stranded overhang on each strand. The sequences of the overhangs were different, and served to anneal the hairpin to the corresponding, complementary single-stranded overhang of each DNA handle (Fig. 1). The DNA handles carried this overhang on one end, and a chemical modification on the opposite end, used to binding a bead, via either an antidigoxigenin-digoxigenin or a biotin-avidin linkage. One handle was 2.7 kbp, with a 25 nt 3′-overhang on one end and a 5′-digoxigenin tag on the opposite end: this was prepared by PCR, templated from a PRL732 plasmid^24^ using a 5′-digoxigenin modified primer, and a primer (sequences provide below) containing an abasic site followed by 25 nt non-complementary sequence that creates a 3′-overhang in the handle during PCR. The second handle was 1 kbp, with a 31 nt 5′-overhang on one end and a 3′-biotin label on the opposite end: this was prepared by PCR templated from a pALB3 plasmid^44^. Sequences of both handles were checked using online database tools (PromoterHunter^45^) to ensure they did not contain cryptic promoter sequences that might interfere with the experiment. To assemble the dumbbell, the hairpin was annealed to the handles in transcription buffer [130 mM Hepes (pH 8.0), 50 mM KCl, 5 mM MgCl_2_, 0.1 mM ethylenediaminetetraacetic acid (EDTA), and 0.1 mM DTT; 26 ± 1 °C] for 45 min, with the hairpin (20 nM) mixed with ~ 4-fold excess of each handle. The annealing mixture was incubated with both anti-digoxigenin-coated 0.9 µm diameter beads and avidin-coated 0.6 µm diameter beads, forming the dumbbell. The resulting dumbbells were diluted 20-fold in transcription buffer and introduced into a flow chamber of ~ 5 μl internal volume. At this stage, the transcription buffer was supplemented by an oxygen-scavenging system [8.3 mg mL^−1^ glucose (Sigma), 46 U mL^−1^ glucose oxidase (Calbiochem), and 94 U mL^−1^ catalase (Sigma)], which is a well-established procedure to protect biomolecules from photo-damage^24^. Catalase and glucose oxidase were purified by FPLC (fast protein liquid chromatography; GE Healthcare) using a Superdex 200 10/300 GL column, and verified to be free of RNase (Ambion RNase Alert).

### Data collection

Our instrument leverages two optical traps formed by dual laser beams that were calibrated using well-established protocols^26^. Uncertainties in force arising from systematic (calibration) errors and to normal variations in bead diameter were estimated to be roughly 15%. To collect pulling data, dumbbell complexes were introduced into a flow chamber (~ 5 μl) together with excess RNAP holoenzyme (105 nM, Epicenter), and in either the presence or absence of nucleotides. The conditions tested were: no NTPs; 1 mM ATP and 1 mM UTP; 1 mM all NTPs (ATP, CTP, GTP, UTP) (Roche); and 1 mM all NTPs with 1 μM Rifampicin (Sigma). Positional data were acquired at a 2 kHz sampling frequency using a suite of custom software (LabVIEW), then filtered at 1 kHz using an 8-pole low-pass Bessel filter, and analyzed offline in Igor Pro (WaveMetrics). Tether extensions and any additional sources of error were determined using established procedures^24^.

To collect real-time initiation data, dumbbell complexes were introduced into the flow chamber in the absence of RNAP holoenzyme. Single dumbbell tethers were trapped and identified, as described^24^. A constant, high load (~ 20 pN) was applied to the tether and data collection was initiated. Under this load, the hairpin is fully unfolded, and the holoenzyme is unable to bind the promoter. Then, ~ 7 µl of buffer containing 105 nM holoenzyme plus 1 mM NTPs was flushed into the flow chamber. After ~ 30 s, the force was lowered to 11–12 pN, a value that is 1–2 pN below the *F*_1/2_ value of the hairpin, where it has a 50% probability of being closed^26, 27^. At this lower load, the double-stranded hairpin reforms, making the promoter DNA available to the RNAP holoenzyme for transcription initiation. Real-time transcription produced a gradual hairpin unzipping that was recorded as an increase in the tether extension, as follows. Upon holoenzyme binding, the region of the DNA upstream of (and unprotected by) the trailing edge of the polymerase is observed to unfold under force. As the holoenzyme loosens and releases its contacts with the promoter, additional hairpin sequences became unprotected. These sequences promptly unzip under the applied load, providing a real-time readout of the progress of initiation (Supplementary Fig. 4a). Subsequent events that generated changes in the upstream binding contacts led to additional increases in tether extension, including the transitions corresponding to promoter escape and productive elongation.

To “walk” the bound RNAP holoenzyme systematically out to different nucleotide positions on the 40DT template, we supplied different subsets of the four NTPs in the buffer (Supplementary Fig. 4a). We began with RNAP holoenzyme in the absence of any NTPs, under which conditions the RNAP holoenzyme remains stationary on the promoter in its RP_o_ state. This led to an initial unzipping of the hairpin out to position –20 ± 3 (Supplementary Fig. 4a). Next, we flowed in 2 mM adenylyl(3′–5′)uridine (ApU) and 1 mM ATP, CTP, and GTP (but no UTP). ApU gets incorporated as a dinucleotide^46^. Under this condition, RNAP synthesizes a transcript of length 29 nt. We observed that the hairpin unzipped until position +20 ± 2 (mean ± S.D.), after displaying pauses at positions –33 ± 2, −24 ± 2, and –10 ± 2. This result indicates that RNAP releases its contacts at positions –33, –24, and –10, escapes the promoter, enters productive elongation, and becomes stalled at position + 29 (i.e., with its trailing edge positioned at ~ + 20). We therefore find that the trailing edge of the RNAP was at position –10 ± 2 when it escaped the promoter and translocated along the template for a distance of 30 ± 3 nt: consistent, within experimental error, with the expected length of transcript (29 nt) produced under this condition. Finally, we introduced the full set of all NTPs into the flow cell, allowing RNAP to continue elongation until reaching the end of the template. We found that the trailing edge moved by an additional 9 ± 3 nt (i.e., to position +29 ± 2) (Supplementary Fig. 4a), consistent, within error, with the RNAP elongation through 11 nt (from positions +29 to +40) until its active site reached the hairpin loop.

### Force stretching curve analysis

FECs were collected by slewing the movable optical trap with an acousto-optic deflector (IntraAction, Inc.) at a fixed rate (190 nm s^−1^), while the position of the bead in the stationary trap was recorded^24^. FECs were collected at a frequency of approximately once every ~ 30 s; sufficient time to allow RNAP holoenzyme rebind to the promoter hairpin between successive pulls.

Unbinding rip sizes were calculated from the difference in contour lengths returned by fits to WLC models, obtained before and after the associated rip^24^. The pre-rip portion of each FEC was fit to a WLC model using a modified Marko-Siggia relationship. Because the pre-rip segment is composed almost entirely of dsDNA, the elastic modulus set to 1200 pN nm^−1^. To ensure single-molecule behavior, we rejected from further analysis any dumbbells exhibiting either an incorrect contour length or too short a persistence length (<18 nm). The post-rip portion of each FEC was fit to a double-WLC model, with the parameters of the first WLC set to those obtained from the pre-rip fit. For the post-rip fit, we assumed a persistence length of 1.0 nm for the single-stranded DNA portion^27, 28^ and an elastic modulus of 1600 pN nm^−1^. We further assumed dsDNA to form a helix of width 2.0 nm, which was subtracted from the extension of the pre-rip portion when fitting FECs.

### Real-time records analysis

Real-time records were truncated to display only those segments acquired under constant (low) loads. Tether extension data were low-pass filtered (end of pass band = 0.1 Hz; start of reject band = 50 Hz, number of coefficients = 500)^21^, and the extension changes were converted to positions along the hairpin relative to the TSS, using established methods^27, 28^. In performing this conversion, the extension under low load (when the hairpin is fully folded) was selected as the reference extension, which was then used as the starting point for real-time records. Pausing positions were calculated by Gaussian fits to the paused regions; only pauses lasting longer than 0.5 s were considered for analysis.

For probability-density plot analysis, records collected in the presence of NTPs from both the 20DT and 40DT templates were trimmed to ~ 2 s before, and ~ 2 s after, the observed initiation events. The mean-density plots in Supplementary Fig. 4b were generated by aggregating density plots, either from *N* = 12 individual records (20DT template) or from *N* = 16 individual records (20DT and 40DT templates), and normalizing the area under the curves. The maxima obtained from the density plots (Fig. 4b) were cross-validated by leaving out one record each time and re-computing the mean-density plot, to ensure that no average peaks arose from a single outlying record.

### Primer sequences

2.7 kbp dsDNA handle PCR primer sequences: primer sequences for the handles were chosen to avoid introducing any cryptic promoter sequences into the DNA handle. A digoxigenin label (“/5DiGN/” in the sequence below) was introduced to the 5′ end of dsDNA handle using the forward primer.

732_handle_dig_fwd sequence:

5′-/5DiGN/GGGTAAAGTGCTGTATAACGCGCGT-3′

732_Pabc_rev sequence:

5′-GGTGTTTCCCCGTGTCCCTCTCGAT/idSp/ACACACACGCCAGTTCCTGAATGTG-3′

1 kbp dsDNA handle PCR primer sequences: a biotin label (“/5BiosG/” in the sequence below) was introduced into the 3′ end of the dsDNA handle through the reverse primer. A phosphorothioate bond (“*”, in the sequence below) was introduced to stop 5′-to-3′ lambda-exonuclease digestion, to create 3′ single-stranded overhang following PCR^44^.

pALB3_3′handle_fwd sequence:

5′-GGTCACCATCATCCTGACTAGAGTCCTTGGC*G-3′

pALB3_rev sequence:

5′-/5BiosG/GTCGTCTTTGCTCAGGATAC-3′.

### Hairpin design

Construction of T7A1_20DT_forward hairpin: the hairpin consists of a T7A1 promoter (36 bp) with a 20 bp upstream sequence, a 20 bp downstream sequence, a downstream tetraloop, and 25 nt single-stranded overhangs separated by an abasic site (labeled “/idSp/” in the sequence below) on either side of the base of the hairpin. The hairpin was constructed by ligating three individual oligonucleotides (IDT). The oligonucleotide sequences are as follows:

T7A1_20DT_forward_oligo1 sequence:

5′-ATCGAGAGGGACACGGGGAAACACC/idSp/AAAATTTATCAAAAAGAGTATTGACTTAAAGTCTAACCTATAGGATACTTACAGCCATCG-3′

T7A1_20DT_forward_oligo2 sequence:

5′-AGAGGGAC ACGGGGAATTTTTTCCC CGTGTC-3′

T7A1_20DT_forward_oligo3 sequence:

5′-CCTCTCGATGGCTGTAAGTATCCTATAGGTTAGACTTTAAGTCAATACTCTTTTTGATAAATTTT/idSp/CATCATCCTGACTAGAGTCCTTGGC-3′.

Construction of T7A1_20DT_reverse hairpin: this hairpin consists of a reversed stem sequence relative to the T7A1_20DT_forward hairpin. In this hairpin, transcription is directed towards the base of hairpin, as opposed to towards the tetraloop. The hairpin was constructed by ligating three individual oligonucleotides (IDT). The oligonucleotide sequences are as follows:

T7A1_20DT_reverse_oligo1 sequence:

5′-ATCGAGAGGGACACGGGGAAACACC/idSp/TTCCCCGTGTCCCTCTCGATGGCTGTAAGTATCCTATAGGTTAGACTTTA-3′

T7A1_20DT_reverse_oligo2 sequence:

5′-AGTCAATACTCTTTTTGATAAATTTTTTTTAAAATTTATCAAAAAGA-3′

T7A1_20DT_reverse_oligo3 sequence:

5′-GTATTGACTTAAAGTCTAACCTATAGGATACTTACAGCCATCGAGAGGGACACGGGGAA/idSp/CATCATCCTGACTAGAGTCCTTGGC-3′.

Construction of T7A1_40DT hairpin: the hairpin was designed identically to the T7A1_20DT_forward hairpin, except that this hairpin carries 40 bp of downstream sequence (instead of 20 bp). The hairpin was constructed by ligating four individual oligonucleotides (IDT). The oligonucleotide sequences are as follows:

T7A1_40DT_oligo1 sequence:

5′-ATCGAGAGGGACACGGGGAAACACC/idSp/AAAATTTATCAAAAAGAGTATTGACTTAAAGTCTAACCTATAGGATACTT-3′

T7A1_40DT_oligo2 sequence:

5′-ACAGCCATCGAGAGGGACACGGGGAAACACCACCATGGTC-3′

T7A1_40DT_oligo3 sequence:

5′-ACCATCTTTTGATGGTGACCATGGTGGTGTTTCCCCGTGTCCCTCTCGATGGCTGTAAGTA-3′

T7A1_40DT_oligo4 sequence:

5′-TCCTATAGGTTAGACTTTAAGTCAATACTCTTTTTGATAAATTTT/idSp/CATCATCCTGACTAGAGTCCTTGGC-3′.

Construction of hairpin for yeast transcription PIC studies: this hairpin consists of the yeast *His4* promoter sequence^38^ from positions −93 to +3. The 32-component PIC comprised of TFIIA, TFIIB, TBP, TFIIE, TFIIH, TFIIF, Sub1, and Pol II. The PIC was purified and assembled from proteins that were either available in recombinant form, or were isolated from yeast^21, 38^. The hairpin was pulled every 5 min, to allow time for the reassembly of the preinitiation complex between successive pulls.

### Potassium permanganate assay

A longer version of T7A1 promoter sequence was used in this assay (250 bp). The sequence of non-template strand sequence is (TSS in bold, −10 element in italics):

5′-CTCACTATAAGGAGAGACAACTTAAAGAGACTTAAAAGATTAATTTAAAATTTATCAAAAAGAGTATTGACTTAAAGTCTAACCTATAGGATACTTACAGCC**A**TCGAGAGGGACACGGGGAAACACCACCATCATCACCATCATCCTGACTAGAGTGCTTGGCGAACCGGTGTTTGACGTCCAGGAATGTCAAATCCGTGGCGTGACCTATTCCGCACCGCTGCGCGTTAAACTGCGTCTGGTGATCTAT-3′.

Two different ^32^P labeling schemes using were used in this assay: 5′ labeling of the non-template strand (upstream labeling), and 5′ labeling of the template strand (downstream labeling). We used the same transcription buffer as in optical-trapping studies. The experimental procedure was described perviously^31^. A concentration of 2 mM of KMnO_4_ was used. The lane compositions (Supplementary Fig. 1) were as follows:

Lane 1: Marker 82 nt

Lane 2: Marker 160 nt

Lane 3: DNA only (labeled upstream end)

Lane 4: DNA + RNAP (labeled upstream end)

Lane 5: DNA only (labeled downstream end)

Lane 6: DNA + RNAP (labeled downstream end).

### Strand-opening-deficient *E. coli* RNAP holoenzyme preparation

Four amino acid residues (FYWW) in the *σ* ^70^ were substituted by alanine^30^. In brief, the *σ* ^70^ mutant was prepared under native conditions through Ni-NTA affinity chromatography, followed by purification on an ion exchange Q-sepharose column. Protein activity was verified by sodium dodecyl sulfate polyacrylamide gel electrophoresis. Proteins (~ 22 μM) were frozen at −80 °C in storage buffer [25 mM Tris-Cl, pH 8.0, 0.1 mM EDTA, 250 mM NaCl, 0.1 mM DTT and 50% glycerol] until use.

### Data availability

The data that support the findings of this study are available from the corresponding author upon request.

## Electronic supplementary material

Supplementary information
