# Supplementary material for: Real-time observation of polymerase-promoter contact remodeling during transcription initiation
Source: Nat Commun. 2017 Oct 27;8:1178. doi: 10.1038/s41467-017-01041-1 (PMC5660091; doi:10.1038/s41467-017-01041-1)
Supplement: Supplementary file 1 — Supplementary information [file 41467_2017_1041_MOESM1_ESM.pdf]

Supplementary Information

Supplementary Figures

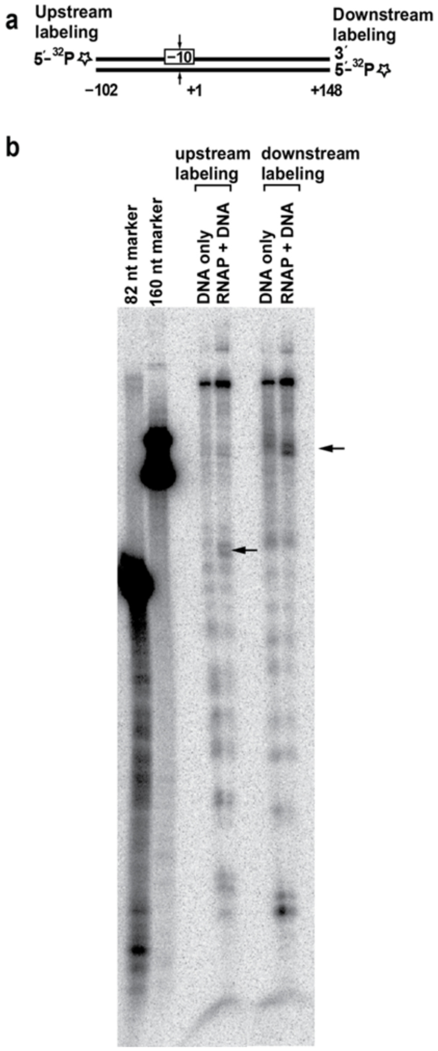

Supplementary Figure 1. $KMnO_4$ assay confirms open-complex formation on the T7A1 promoter. (a) Schematic of the sequence used in the $KMnO_4$ protection assay, which maps single-stranded regions formed during initiation. The length of the DNA is 250 bp, extending from −102 to +148 of the T7A1 promoter. Two labeling schemes with ^{32}P were used in the assay: (1) upstream labeling, on the 5′ -end of the non-template strand, and (2) downstream labeling, on the 5′ -end of the template strand. The −10 hexameric sequence is indicated (square box). Black arrows show the positions on DNA accessible to $KMnO_4$, as identified by this experiment. (b) Electrophoretic gel of the $KMnO_4$ digestion. Two lengths of markers were used: 82 nt and 160 nt. Each of the two labeled DNA templates were treated with $KMnO_4$, with and without prior incubation with RNAP holoenzyme (lanes labeled). Locations accessible to $KMnO_4$ specifically in the presence of holoenzyme contacts are indicated (black arrows). The left arrow corresponds to a product of ~90 nt generated from the non-template strand; the right arrow corresponds to a product of ~160 nt. Both products indicate increased accessibility on template and non-template strands to $KMnO_4$ around the −10 element.

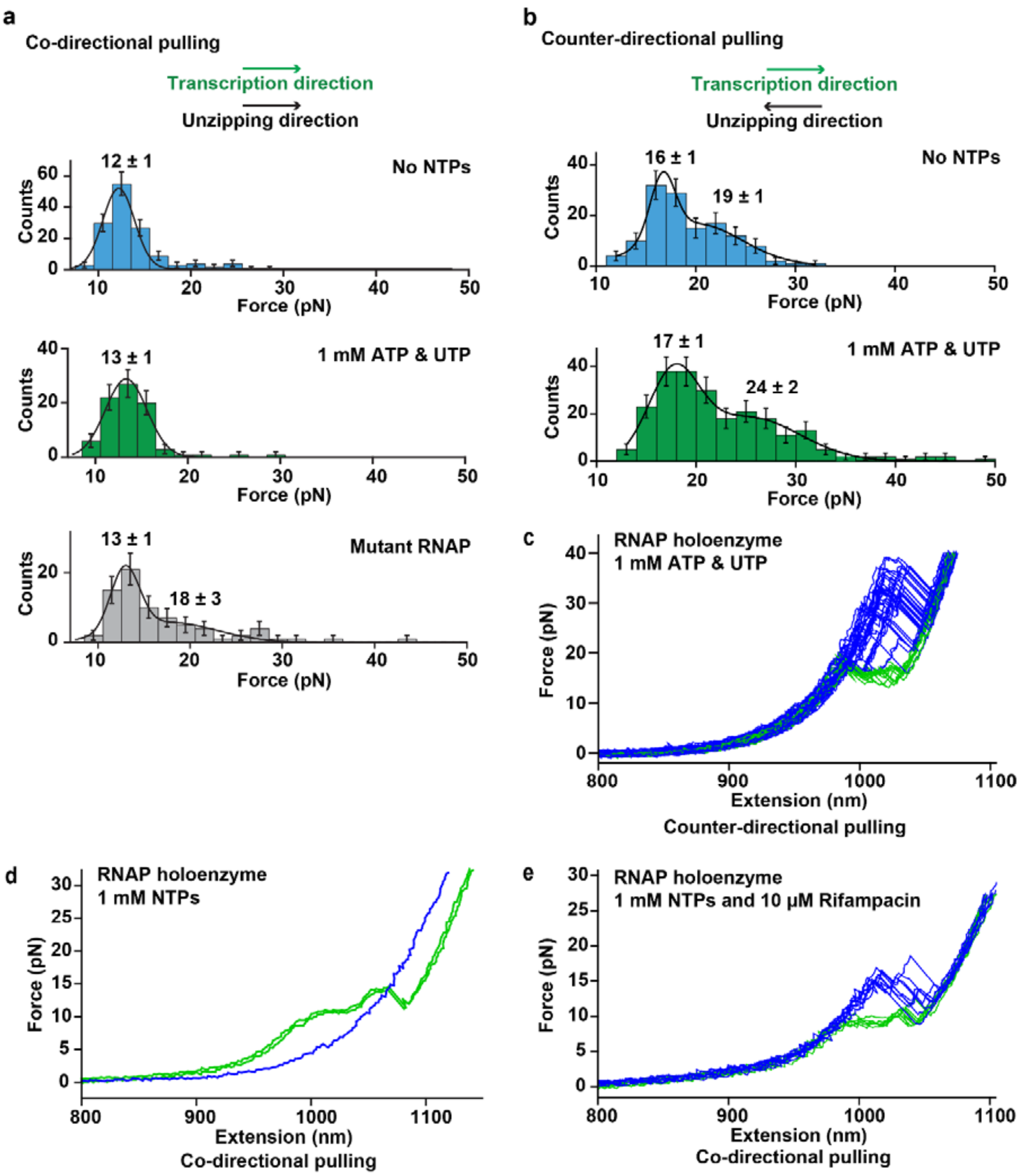

Supplementary Figure 2. Hairpin opening-force distributions, for both (a) co-directional and (b) counter-directional pulling on 20DT with RNAP holoenzyme present. (a) Histograms of co-directional opening forces for the wild-type RNAP with no NTPs (blue; $N = 139$), and with 1 mM ATP, UTP (green; $N = 82$); data from the strand opening-deficient mutant are shown in black ($N = 76$). (b) Histograms of counter-directional opening forces from wild-type RNAP with no NTPs (blue; $N = 131$), and with 1 mM ATP, UTP (green; $N = 231$). (c) Representative FECs of the hairpin with RNAP holoenzyme bound and ATP, UTP present. FECs of the 20DT hairpin when (d) RNAP and all NTPs were present (blue curves), and when (e) RNAP holoenzyme, 1 mM NTPs and 10 µM rifampicin were present (blue curves). FECs of the 20DT hairpin with no RNAP present (green curves).

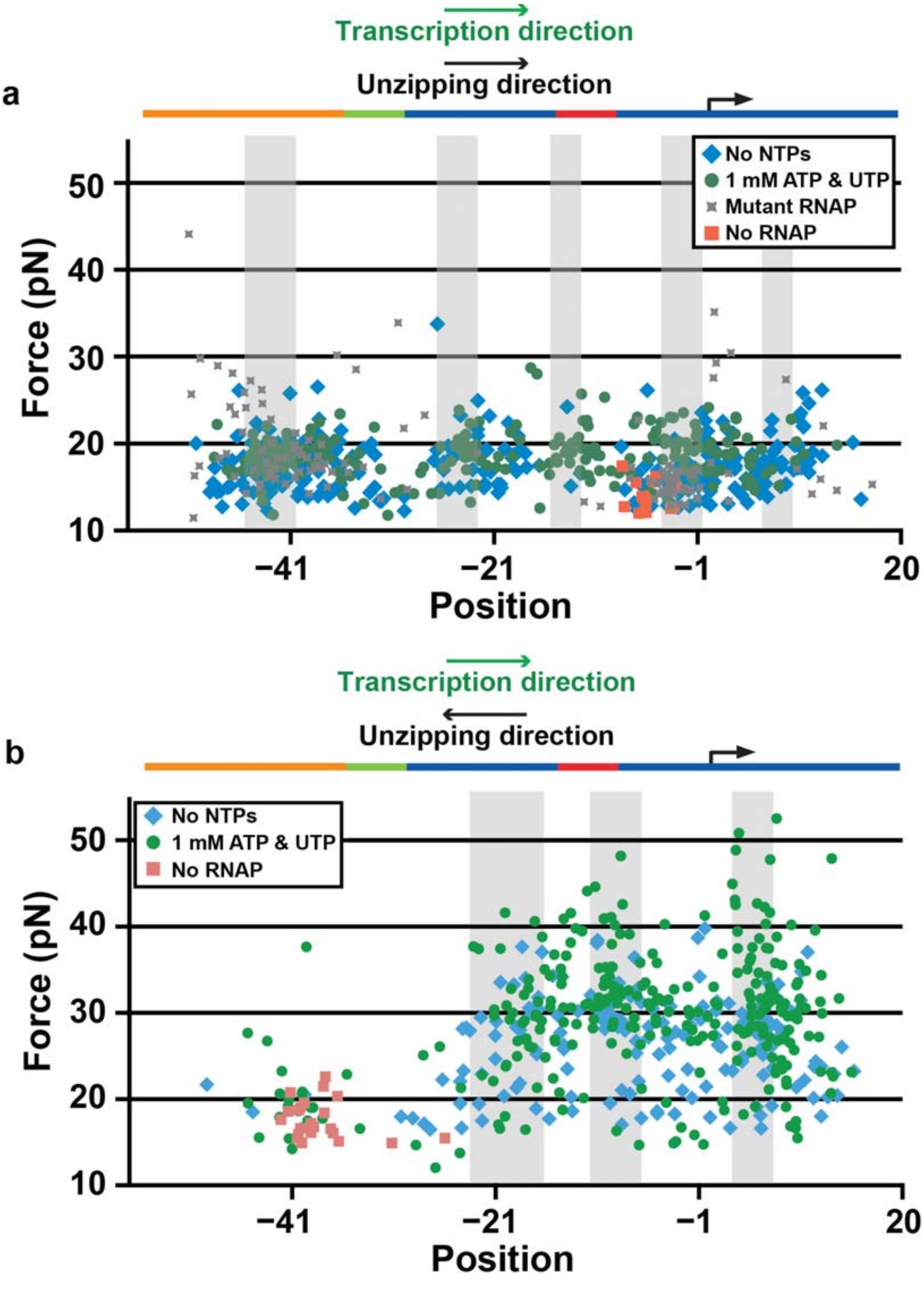

Supplementary Figure 3. 2D histogram of the force of dissociation of various contacts in both the (a) co-directional and (b) counter-directional pulling for the following conditions: (1) RNAP with no NTPs added (blue diamond); (2) RNAP with 1mM ATP and UTP added (green circle); (3) bare hairpin, with no RNAP added (orange square); and (4) mutant RNAP with no NTPs added (black cross). The grey regions show the fit mean for the various contacts observed, along with errors.

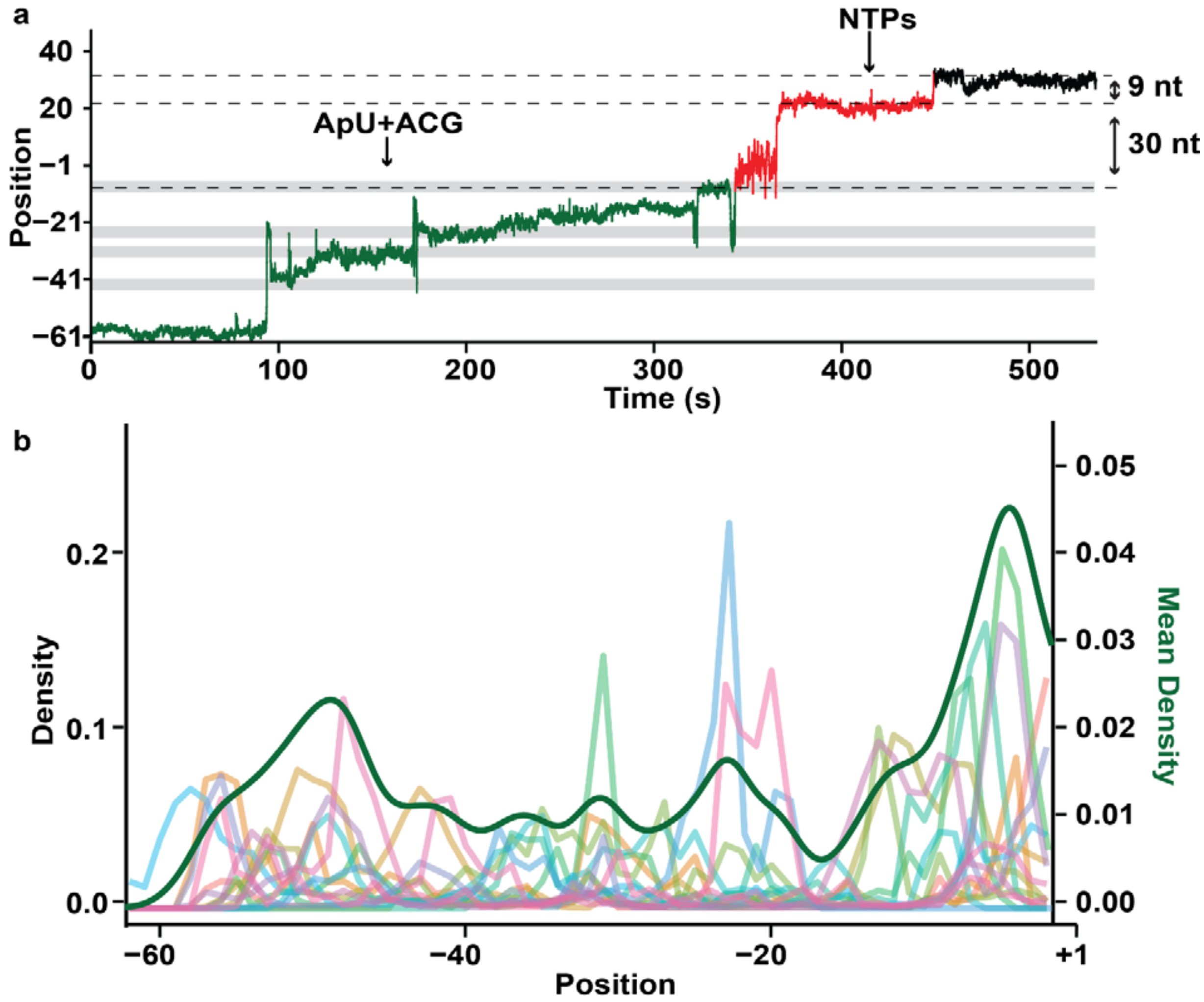

Supplementary Figure 4 Real-time transcription initiation in the presence of NTPs. (a) A record showing transcription initiation as the medium was exchanged in the flow-cell: (1) ~105 nM RNAP holoenzyme (introduced before t = 0 s); followed by (2) 2 mM ApU and 1 mM ATP, CTP, GTP (left vertical arrow); followed by (3) 1 mM NTPs (right vertical arrow). (b) Probability density plots of individual records of transcription initiation collected on the 20DT and 40DT templates in the presence of NTPs (N = 16, light colors), with the average density superposed (dark green).

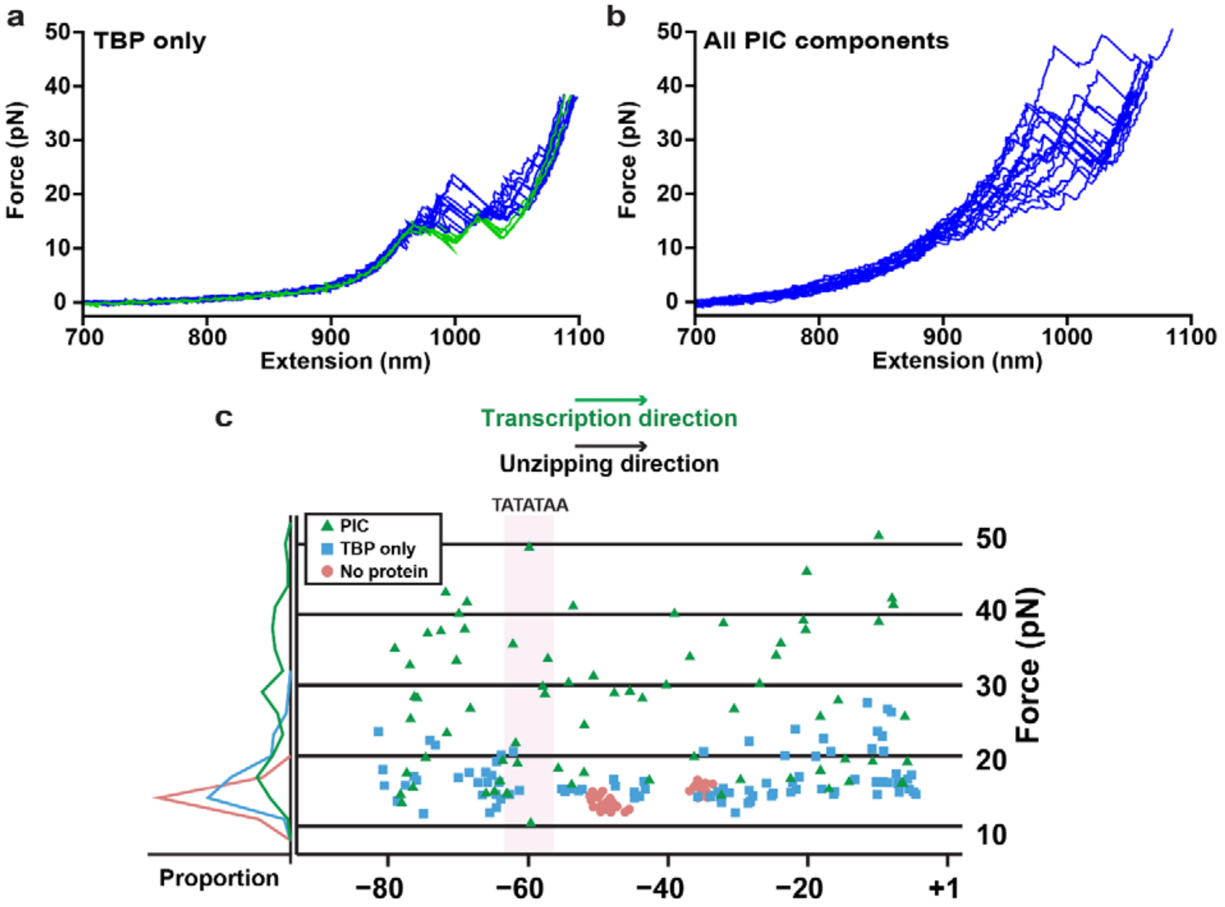

Supplementary Figure 5. Force-extension curves of a hairpin carrying the yeast His4 promoter (a) when TATA-binding protein (TBP) is present (TBP-dependent FECs shown in blue, and FECs with no protein bound shown in green) and (b) when all 32-protein components of the PIC are present, including the transcription factors TFIIA, TFIIB, TBP, TFIIE, TFIIF and TFIIH. (c) 2D histogram of the dissociation force for various contacts when either TBP (blue squares) or the full PIC (green triangles) are added, relative to the bare hairpin (no protein; orange squares). The pink area shows the location of the TATATAA sequence. Both TBP and PIC show extensive contacts along the promoter DNA.
